# Complex Sepsis Presentations, SEP-1 Compliance, and Outcomes

**DOI:** 10.1001/jamanetworkopen.2025.1100

**Published:** 2025-03-19

**Authors:** Chanu Rhee, Sarah E. Train, Michael R. Filbin, Steven T. Park, Nicholas M. Mohr, Anne Zepeski, Brett A. Faine, David J. Roach, Emily Porter, Claire N. Shappell, Kamryn Plechot, Laura DelloStritto, Tingting Yu, Michael Klompas

**Affiliations:** 1 Department of Population Medicine, Harvard Pilgrim Health Care Institute, Boston, Massachusetts; 2 Division of Infectious Diseases, Department of Medicine, Brigham and Women’s Hospital, Boston, Massachusetts; 3 Division of Pulmonary and Critical Care Medicine, Department of Medicine, Brigham and Women’s Hospital, Boston, Massachusetts; 4 Division of Pulmonary and Critical Care Medicine, Department of Medicine, University of Wisconsin–Madison School of Medicine and Public Health, Madison; 5 Department of Emergency Medicine, Massachusetts General Hospital, Boston; 6 Division of Infectious Diseases, Department of Medicine, University of California, Irvine, School of Medicine, Orange; 7 Department of Emergency Medicine, University of Iowa Health Care, Iowa City; 8 Department of Emergency Medicine, University of California Medical Center, Orange

## Abstract

**Importance:**

The Centers for Medicare & Medicaid Services Severe Sepsis and Septic Shock Management Bundle (SEP-1) is supported by observational studies that report SEP-1 compliance is associated with lower mortality. Most studies, however, adjusted for limited confounders and provided little insight into why bundle-compliant care was not provided.

**Objectives:**

To identify the clinical factors that complicate the diagnosis and management of sepsis and assess their association with SEP-1 compliance and mortality.

**Design, Setting, and Participants:**

This retrospective cohort study was conducted among 590 adults with sepsis in the emergency department of 4 academic hospitals from January 1, 2019, to December 31, 2022. Patients’ medical records were reviewed between September 2022 and December 2023.

**Main Outcomes and Measures:**

Study outcomes were (1) characteristics of patients who received SEP-1–compliant care vs characteristics of patients who received noncompliant care and (2) association between SEP-1 compliance and hospital mortality using multivariable models to adjust for successively more potential confounders (first demographics and comorbidities, then infection source, then severity of illness, and then clinical markers of complexity).

**Results:**

Of 590 patients with sepsis (median age, 65 years [IQR, 53-77 years]; 329 men [55.8%]), 335 (56.8%) received SEP-1–compliant care, and 225 (43.2%) received noncompliant care. Compared with patients in the compliant group, patients in the noncompliant group were more likely to be 65 years or older (142 [55.7%] vs 158 [47.2%]; odds ratio [OR], 1.41 [95% CI, 1.01-1.95]), to have multiple comorbidities (Elixhauser score >20: 99 [38.8%] vs 99 [29.6%]; OR, 1.51 [95% CI, 1.07-2.13]), and to have a higher incidence of septic shock (107 [42.0%] vs 107 [31.9%]; OR, 1.54 [95% CI, 1.10-2.16]), kidney dysfunction (87 [34.1%] vs 80 [23.9%]; OR, 1.65 [95% CI, 1.15-2.37]), and thrombocytopenia (43 [16.9%] vs 37 [11.0%]; OR, 1.16 [95% CI, 1.02-2.62]) on presentation. Compared with patients in the compliant group, those in the noncompliant group also had more nonfebrile presentations (136 [53.3%] vs 121 [36.1%]; OR, 2.02 [95% CI, 1.45-2.82]), impaired mental status (92 [36.1%] vs 94 [28.1%]; OR, 1.45 [95% CI, 1.02-2.05]), need for bedside procedures (57 [22.4%] vs 41 [12.2%]; OR, 2.06 [95% CI, 1.33-3.21]), acute concurrent noninfectious illnesses (140 [54.9%] vs 151 [45.1%]; OR, 1.48 [95% CI, 1.07-2.06]), and noninfectious illness as the primary factor associated with their presentation (84 [32.9%] vs 71 [21.2%]; OR, 1.82 [95% CI, 1.08-3.08]). SEP-1 compliance was associated with lower crude mortality rates compared with noncompliance (40 [11.9%] vs 41 [16.1%]; unadjusted OR, 0.60 [95% CI, 0.37-0.98]), but there was no statistically significant difference between groups after successively adjusting for demographics and comorbidities (adjusted OR [AOR], 0.71 [95% CI, 0.42-1.18]), infection source (AOR, 0.71 [95% CI, 0.43-1.20]), severity of illness (AOR, 0.86 [95% CI, 0.50-1.49]), and clinical markers of complexity (AOR, 1.08 [95% CI, 0.61-1.91]).

**Conclusions and Relevance:**

In this cohort study of adults with sepsis, complex clinical presentations were more common among patients whose treatment was noncompliant with SEP-1. These nuances are poorly captured in most observational studies but confound the association between SEP-1 compliance and mortality.

## Key Points

**Question:**

Are sepsis presentations associated with Severe Sepsis and Septic Shock Management Bundle (SEP-1) compliance and mortality?

**Findings:**

In this cohort study of 590 patients with sepsis, noncompliance was more common among patients with high comorbidity burden presenting with nonfebrile symptoms, impaired mental status, concurrent acute noninfectious conditions, and septic shock, as well as among patients who required bedside procedures. After adjustment for these and other patient characteristics, the association between SEP-1 compliance and mortality changed from protective to null.

**Meaning:**

This study suggests that complex presentations and management barriers are more common among patients who receive care that is noncompliant with SEP-1 and likely confound the association between SEP-1 compliance and mortality.

## Teaser

This cohort study identifies the clinical factors that complicate the diagnosis and management of sepsis and assesses their association with Severe Sepsis and Septic Shock Management Bundle (SEP-1) compliance and mortality.

## Introduction

Sepsis is a leading cause of death, disability, and resource utilization.^1,2^ The Centers for Medicare & Medicaid Services (CMS) seeks to improve sepsis outcomes by requiring hospitals to report their compliance with the Severe Sepsis and Septic Shock Management Bundle (SEP-1).^3^ SEP-1 was based on the Surviving Sepsis Campaign bundle and supported by studies reporting that bundle compliance is associated with lower mortality rates.^4,5,6,7,8,9,10^ CMS is currently transitioning SEP-1 from pay for reporting to pay for performance.^11^

There is substantial controversy, however, over the true impact of the SEP-1 bundle.^12,13,14,15^ Stakeholders have pointed out that observational studies comparing outcomes among SEP-1–compliant vs SEP-1–noncompliant groups are at high risk of confounding.^11,12^ For example, patients with septic shock are less likely than those without shock to receive SEP-1–compliant care, in part because SEP-1 requires more steps for patients with shock. Patients with septic shock, however, have higher mortality rates, leading to a crude association between SEP-1 compliance and lower mortality.^16^ Similarly, older patients and those with cancer and other serious comorbidities are less likely to receive SEP-1–compliant care, but these conditions are also independently associated with higher mortality (regardless of whether patients have septic shock or sepsis alone).^17,18,19,20,21,22^

Given the heterogeneity of sepsis presentations, there may be additional diagnostic and therapeutic factors associated with SEP-1 bundle compliance and outcomes.^23^ Most previous studies of bundle compliance have relied on administrative and/or electronic health record (EHR) data, limiting their ability to identify complex clinical factors that are not usually captured in structured fields (such as clinicians’ initial level of suspicion for infection, difficult intravenous access, need for urgent bedside procedures, and competing noninfectious diagnoses). Identifying these factors could help highlight important barriers to timely sepsis care and provide a better understanding of the association of bundle compliance with outcomes. We therefore undertook detailed medical record reviews to describe the characteristics of patients who did and patients who did not receive SEP-1–compliant care, with a particular focus on clinical factors that are difficult to identify in administrative or structured electronic clinical data. We then assessed how associations between SEP-1 compliance and mortality changed when adding these potential confounders into multivariable models.

## Methods

### Study Design, Setting, Patients, and Summary of SEP-1 Specifications

This was a retrospective cohort study of adults abstracted for SEP-1 at 4 academic teaching hospitals in Massachusetts (Brigham and Women’s Hospital and Massachusetts General Hospital), Iowa (University of Iowa Hospital & Clinics), and California (University of California, Irvine Medical Center) between January 1, 2019, and December 31, 2022. As per CMS requirements, staff at each hospital abstracted up to 20 cases per month with *International Statistical Classification of Diseases and Related Health Problems, Tenth Revision* (*ICD-10*) codes for sepsis to assess whether they met SEP-1 criteria for severe sepsis (documentation of suspected infection, ≥2 systemic inflammatory response syndrome criteria, and acute organ dysfunction within 6 hours, with time zero defined as the moment the last criterion was met) or septic shock (severe sepsis plus persistent hypotension despite ≥30 mL/kg of fluids, initial lactate level ≥4.0 mmol/L, or documentation of possible septic shock) and whether the 3- and 6-hour bundles were completed per SEP-1 specifications.^10^ Patients with severe sepsis require the 3-hour bundle (lactate measurement, blood cultures, antibiotic administration, and 30 mL/kg fluids for hypotension or lactate level ≥4.0 mmol/L) plus a repeat lactate level by 6 hours if the initial lactate level was greater than 2.0 mmol/L; those with septic shock further require vasopressors for persistent hypotension and documentation of a repeat volume or perfusion status examination within 6 hours. SEP-1 is an “all-or-nothing” measure that allows abstractors to stop once any bundle component is found to be noncompliant.

We randomly selected up to 150 cases per hospital (from among those that the hospital had submitted to CMS) for detailed medical record review. We limited our study to patients who met SEP-1 time zero criteria in the emergency department (ED) because most sepsis cases are community onset and the ED is a more homogeneous setting than inpatient units.^19^

Reporting followed the Strengthening the Reporting of Observational Studies in Epidemiology (STROBE) reporting guideline for cohort studies.^24^ The institutional review boards of each site approved the study with a waiver of informed consent given the high mortality rates of patients with sepsis included in the study, making collection of consent infeasible.

### Medical Record Review Process

We used a standardized abstraction tool in REDCap to record patients’ demographic characteristics, encounter characteristics, outcomes, discharge diagnosis codes, SEP-1 compliance and first failed bundle element (for noncompliant cases), initial vital signs and laboratory test results, and detailed information about their clinical presentation and ED course (eAppendix in Supplement 1).^25^ Data related to patients’ clinical presentation and ED course were all manually abstracted by clinician reviewers (S.E.T., M.R.F., S.T.P., A.Z., B.A.F., D.J.R., E.P., C.N.S., and K.P.) (eMethods in Supplement 1). Medical record reviews were conducted between September 2022 and December 2023.

We focused on identifying clinical markers of complexity that might be associated with the time to diagnosis and therapeutic management but are difficult to capture in administrative and EHR datasets. Specific items to abstract were determined by consensus among the investigative team, informed by clinical experience and prior literature. These factors included (1) impaired mental status (including alcohol or drug intoxication, aggressive behavior, delirium, or dementia); (2) non-English primary language; (3) difficult intravenous access (documentation of multiple placement attempts, need for ultrasonography-guided placement, or intraosseous or central venous catheter placement); (4) presenting symptoms suggestive of infection, including subjective fevers, chills, or rigors and other explicit infectious symptoms (eg, productive cough, dysuria, skin erythema or wound drainage, referral to the ED for suspected infection);^17^ (5) whether infection or sepsis was documented as the leading initial diagnosis by ED clinicians; (6) identification of a clear infection source by the time of ED discharge; (7) presence of heart failure or end-stage kidney disease or documented concern for volume overload; (8) do-not-resuscitate or do-not-intubate status or other care limitations on arrival; (9) need for subspecialty consultation in the ED; (10) computed tomography (CT) scans in the ED; (11) bedside procedures in the ED (eg, central venous catheter, intubation, thoracentesis, lumbar puncture, paracentesis, bronchoscopy); and (12) whether other acute nonbacterial or noninfectious conditions (eg, pulmonary embolism, heart failure, hemorrhage) contributed to the patient’s presenting illness using all available information from the patient’s hospitalization, and, if so, whether in hindsight an infectious or noninfectious process was the primary factor associated with the patient’s presentation.

### Statistical Analysis

We performed descriptive analyses comparing the characteristics of patients who received SEP-1–compliant vs SEP-1–noncompliant care using the Wilcoxon rank sum test for continuous variables and the χ^2^ statistic for categorical variables. Characteristics were binned into 4 categories based on data complexity: (1) baseline characteristics (eg, demographics including race [White, Black, and other (Asian, American Indian or Alaska Native, Native Hawaiian or Other Pacific Islander, ≥2 races, and unknown)] as reported by patients and recorded in each hospital’s EHR database, given that prior studies have reported an association of race and ethnicity with sepsis outcomes^26,27^; comorbidities derived using *ICD-10* discharge diagnosis codes as per the Agency for Healthcare Research and Quality [AHRQ] Elixhauser mortality index [version 2024.1]^28^; and encounter-level information); (2) source of infection; (3) severity-of-illness variables based on initial vital signs (temperature ≥38.0 °C, systolic blood pressure <90 mm Hg, respiratory rate ≥22 breaths/min, oxygen saturation ≤92%) and laboratory test results (lactate >2.0 mmol/L, creatinine ≥2.0 mg/dL [to convert to micromoles per liter, multiply by 88.4], bilirubin ≥2.0 mg/dL [to convert to micromoles per liter, multiply by 17.104], platelet count <100 × 10^3^/µL [to convert to cells ×10^9^/L, multiply by 1.0], and white blood cell count >12 000 cells/µL or <4000 cells /µL [to convert to cells ×10^9^/L, multiply by 0.001]), as well as septic shock; and (4) clinical markers of complexity (as already defined) that may have slowed diagnosis or delayed therapeutic management. For the severity-of-illness category, we defined septic shock using physiological criteria (persistent hypotension requiring vasopressors or initial lactate level ≥4.0 mmol/L) rather than SEP-1 criteria because the latter allows subjective documentation (ie, clinician documentation of suspected septic shock) to qualify even in the absence of objective evidence of shock and misses patients with vasopressor-dependent hypotension if less than 30 mL/kg of fluids were administered within 3 hours. There were no missing data for vital signs; missing laboratory values were assumed to be normal.

To assess the association of adding more detailed clinical data with bundle compliance (the exposure of interest) and in-hospital mortality (the outcome of interest), we constructed multivariable logistic models with hospital-specific intercepts across 4 hierarchies by incrementally adding covariates from the different categories. All multivariable models were selected based on the bayesian information criteria (BIC) using a forward-backward stepwise search algorithm. The algorithm searches between a specified minimal model, which is the model selected in the previous hierarchy, and a specified maximal model, which consists of the previously selected model plus all covariates in the new category. At each hierarchy level, the subset of covariates yielding the smallest BIC was selected for the multivariable model. We calculated generalized variance inflation factors (gVIFs) to assess multicollinearity among the covariates. We also used type II and type III analysis of variance to test the significance of main effects and interaction effects among the variables selected in the final model.

We subsequently stratified these models for patients with septic shock (defined using physiological criteria) vs severe sepsis alone. We also performed a sensitivity analysis using a combined outcome of in-hospital death, discharge to hospice, or intensive care unit (ICU) length of stay of 3 days or more. All tests of significance were 2-sided, and results were deemed statistically significant at *P* < .05. Analyses were conducted using SAS, version 9.4 (SAS Institute Inc) and R, version 4.2.1 (R Project for Statistical Computing).

## Results

### Study Cohort and Reasons for SEP-1 Failure

**Figure 1.**
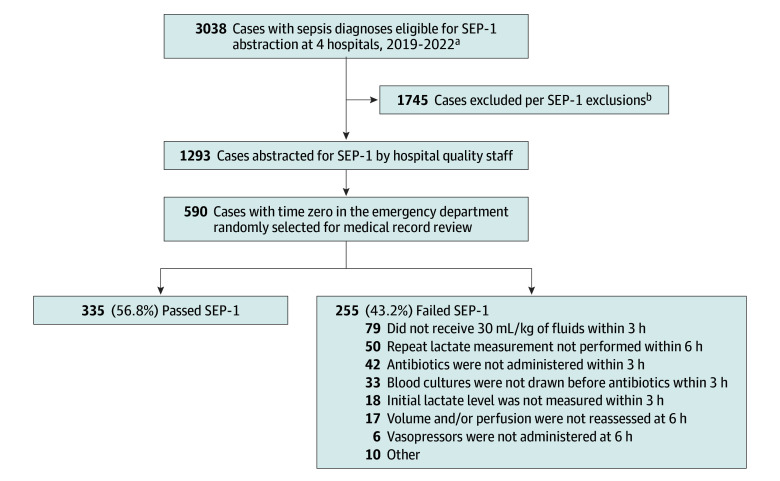
Study Flowchart SEP-1 indicates Severe Sepsis and Septic Shock Management Bundle. ^a^Cases from 3 of the hospitals were selected from 2019 to 2021; cases from the fourth hospital were selected from 2020 to 2022. ^b^Most cases were commonly transferred from outside the hospital or did not meet the Centers for Medicare & Medicaid Services time zero criteria.

The analytic cohort included 590 patients (median age, 65 years [IQR, 53-77 years]; 329 men [55.8%] and 261 women [44.2%]; 43 Black patients [7.3%], 439 White patients [74.4%], and 108 patients of other race [18.3%]), of whom 335 (56.8%) received SEP-1–compliant care and 255 (43.2%) received SEP-1–noncompliant care (Figure 1 and Table). Comorbidities were common (median AHRQ Elixhauser score, 12 [IQR, 0-26]), 216 patients (36.6%) were admitted to the ICU, the median hospital length of stay was 8 days (IQR, 5-12 days), and 81 patients (13.7%) died in the hospital (Table). A total of 214 patients (36.3%) had septic shock as defined by vasopressor-dependent hypotension or a lactate level of 4.0 mmol/L or more. SEP-1 compliance rates and outcomes across the 4 hospitals are presented in eTable 1 in Supplement 1. Within the SEP-1–noncompliant group, the most common reasons for bundle failure were not administering 30 mL/kg of fluids as required within 3 hours for patients with hypotension or a lactate level of 4.0 mmol/L or more (79 of 255 [31.0%]), not repeating lactate measurements within 6 hours after an initial elevated lactate level (50 of 255 [19.6%]), and not administering antibiotics within 3 hours (42 of 255 [16.5%]) (Figure 1).

**Table.** Baseline Patient Characteristics and Outcomes of Patients With Sepsis

Characteristic	All patients with sepsis (N = 590)	Group		*P* value
		SEP-1 compliant (n = 335)	SEP-1 noncompliant (n = 255)	
Age, median (IQR), y	65 (53-77)	63 (52-75)	67 (54-78)	.05
Sex, No. (%)				
Female	261 (44.2)	147 (43.9)	114 (44.7)	.84
Male	329 (55.8)	188 (56.1)	141 (55.3)	
Race, No. (%)				
Black	43 (7.3)	26 (7.8)	17 (6.7)	.35
White	439 (74.4)	244 (72.8)	195 (76.5)	
Other^a^	108 (18.3)	65 (19.4)	43 (16.9)	
Select comorbidities, No. (%)				
Chronic lung disease	99 (16.8)	53 (15.8)	46 (18.0)	.48
Congestive heart failure	134 (22.7)	69 (20.6)	65 (25.5)	.16
Diabetes^b^	153 (25.9)	72 (21.5)	81 (31.8)	.005
Leukemia or lymphoma	37 (6.3)	23 (6.9)	14 (5.5)	.50
Liver disease	62 (10.5)	33 (9.9)	29 (11.4)	.55
Neurologic disease	100 (17.0)	49 (14.6)	51 (20.0)	.09
Solid cancer^c^	70 (11.9)	39 (11.6)	31 (12.2)	.85
Kidney disease	140 (23.7)	72 (21.5)	68 (26.7)	.14
AHRQ Elixhauser score, median (IQR)	12 (0-26)	10 (0-23)	14 (0-30)	.04
Admission from facility or hospice, No. (%)	59 (10.0)	39 (11.6)	20 (7.8)	.13
Arrival via emergency medical services, No. (%)	267 (45.3)	150 (44.8)	117 (45.9)	.79
Hospitalization, past 90 d, No. (%)	243 (41.2)	141 (42.1)	102 (40.0)	.61
ICU admission (from ED), No. (%)	216 (36.6)	112 (33.4)	104 (40.8)	.07
Admitting service, No. (%)				
Medical	529 (89.7)	303 (90.5)	226 (88.6)	.59
Surgical	53 (9.0)	29 (8.7)	24 (9.4)	
Other	8 (1.4)	3 (0.9)	5 (2.0)	
ICU length of stay, median (IQR), d	3 (2-6)	3 (2-6)	3 (2-6)	.57
Hospital length of stay, median (IQR), d	8 (5-12)	7 (5-11)	8 (5-13)	.26
Outcomes, No. (%)				
In-hospital death	81 (13.7)	40 (11.9)	41 (16.1)	.15
Discharge to hospice	38 (6.4)	22 (6.6)	16 (6.3)	.89

Abbreviations: AHRQ, Agency for Healthcare Research and Quality; ED, emergency department; ICU, intensive care unit; SEP-1, Severe Sepsis and Septic Shock Management Bundle.

^a^ Includes Asian, American Indian or Alaska Native, Native Hawaiian or Other Pacific Islander, 2 or more races, and unknown.

^b^ Includes diabetes with or without complications.

^c^ Includes solid tumor with or without metastases.

### Comparison of Baseline Characteristics, Sources of Infection, and Severity of Illness

**Figure 2.**
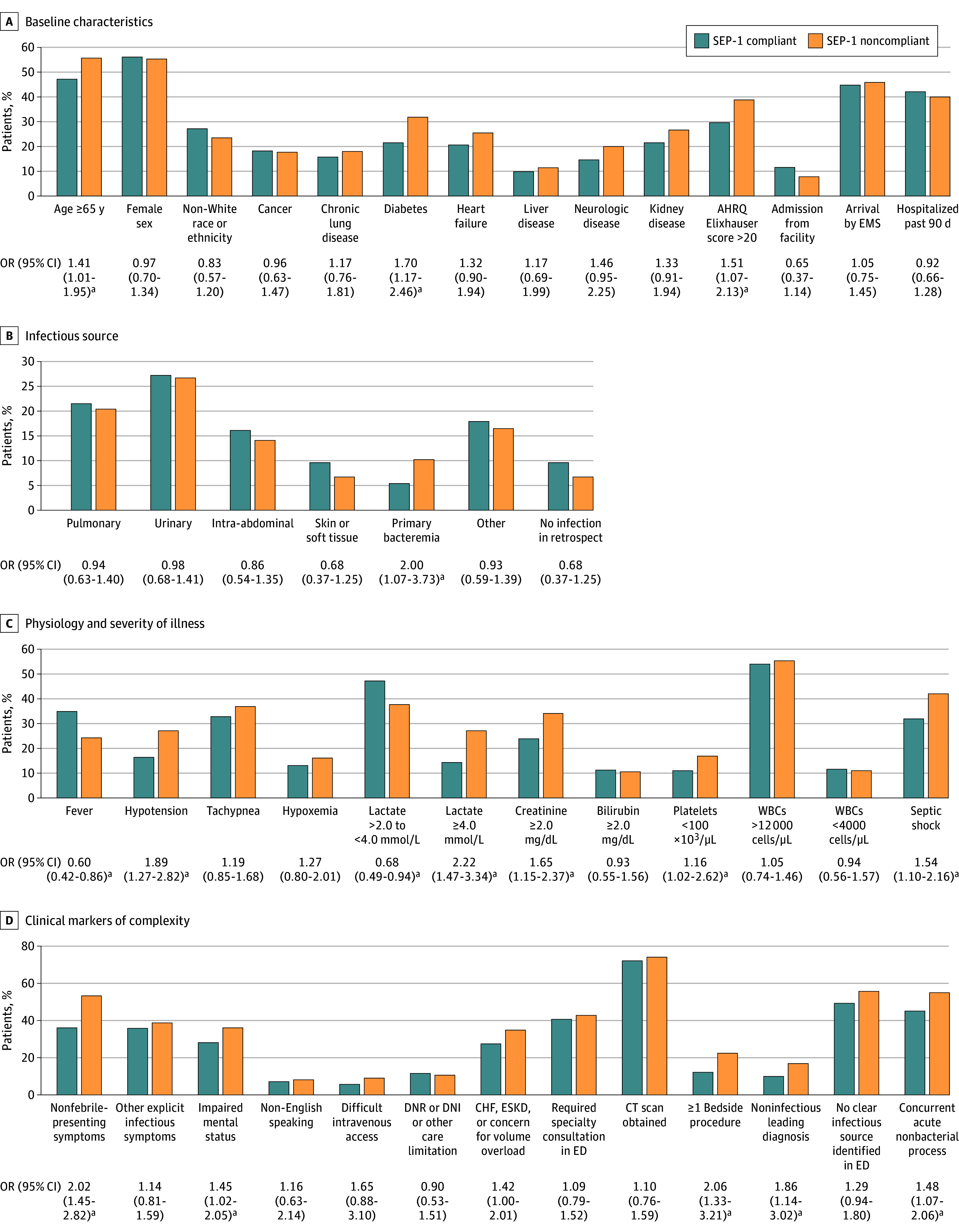
Comparison of Baseline and Clinical Characteristics in SEP-1–Compliant vs SEP-1–Noncompliant Groups SI conversion factors: To convert creatinine to micromoles per liter, multiply by 88.4; bilirubin to micromoles per liter, multiply by 17.104; platelets to cells ×10^9^/L, multiply by 1.0; and WBCs to cells ×10^9^/L, multiply by 0.001. ^a^Statistically significant differences between the 2 groups.

Patients in the noncompliant group were more likely to be 65 years or older (142 [55.7%] vs 158 [47.2%]; odds ratio [OR], 1.41 [95% CI, 1.01-1.95]), to have diabetes (81 [31.8%] vs 72 [21.5%]; OR, 1.70 [95% CI, 1.17-2.46]), and to have multiple comorbidities (AHRQ Elixhauser score >20: 99 [38.8%] vs 99 [29.6%]; OR, 1.51 [95% CI, 1.07-2.13]) (Figure 2A). The groups were otherwise similar in terms of sex, White vs race other than White, admission from a health care facility, arrival to the ED via ambulance, and hospitalization within the preceding 90 days. Sources of infection were also similar, with the exception of more primary bacteremia in the SEP-1–noncompliant group (26 [10.2%] vs 18 [5.4%]; OR, 2.00 [95% CI, 1.07-3.73]) (Figure 2B).

Rates of missing laboratory test values were highest for bilirubin (36 [6.1%]) and low for other values (platelets, 12 [2.0%]; lactate, 6 [1.0%]; white blood cell count, 5 [0.8%]; and creatinine, 3 [0.5%]). Patients who received SEP-1–noncompliant care were less likely to present with objective fever (62 [24.3%] vs 117 [34.9%]; OR, 0.60 [95% CI, 0.42-0.86]) and more likely to have septic shock (107 [42.0%] vs 107 [31.9%]; OR, 1.54 [95% CI, 1.10-2.16]), kidney dysfunction (creatinine ≥2.0 mg/dL; 87 [34.1%] vs 80 [23.9%]; OR, 1.65 [95% CI, 1.15-2.37]), and thrombocytopenia (platelet count <100 × 10^3^/µL; 43 [16.9%] vs 37 [11.0%]; OR, 1.16 [95% CI, 1.02-2.62]) (Figure 2C). There were no significant differences in tachypnea, hypoxemia, hyperbilirubinemia, leukocytosis, or leukopenia.

### Clinical Markers of Complexity

Patients in the SEP-1–noncompliant group were more likely to present without a subjective history of fever, rigors, or chills (136 [53.3%] vs 121 [36.1%]; OR, 2.02 [95% CI, 1.45-2.82]) but had similar rates for other explicit infectious symptoms (99 [38.8%] vs 120 [35.8%]; OR, 1.14 [95% CI, 0.81-1.59]) (Figure 2D). They more frequently had impaired mental status (92 [36.1%] vs 94 [28.1%]; OR, 1.45 [95% CI, 1.02-2.05]), required a bedside procedure (57 [22.4%] vs 41 [12.2%]; OR, 2.06 [95% CI, 1.33-3.21]), had ED clinicians consider noninfectious conditions at the top of their differential diagnosis (43 [16.9%] vs 33 [9.9%]; OR, 1.86 [95% CI, 1.14-3.02]; had concurrent acute nonbacterial or noninfectious processes (140 [54.9%] vs 151 [45.1%]; OR, 1.48 [95% CI, 1.07-2.06]); and had acute nonbacterial or noninfectious processes as the primary factor associated with their presentation (84 [32.9%] vs 71 [21.2%]; OR, 1.82 [95% CI, 1.08-3.08]). There were no significant differences in the other examined markers of clinical complexity.

The most common ED bedside procedures were central venous catheter placement (47 [8.0%]) and intubation (34 [5.8%]) (eFigure 1 in Supplement 1). Among the 291 patients who had concurrent acute nonbacterial or noninfectious processes associated with their presentation, the most common illnesses were new or progressive cancer (51 [17.5%]), heart failure or cardiogenic shock (38 [13.1%]), electrolyte abnormalities (27 [9.3%]), arrhythmias (25 [8.6%]), and chronic lung disease exacerbations (15 [5.2%]) (eFigure 2 in Supplement 1).

### Association Between SEP-1 Compliance and In-Hospital Mortality

**Figure 3.**
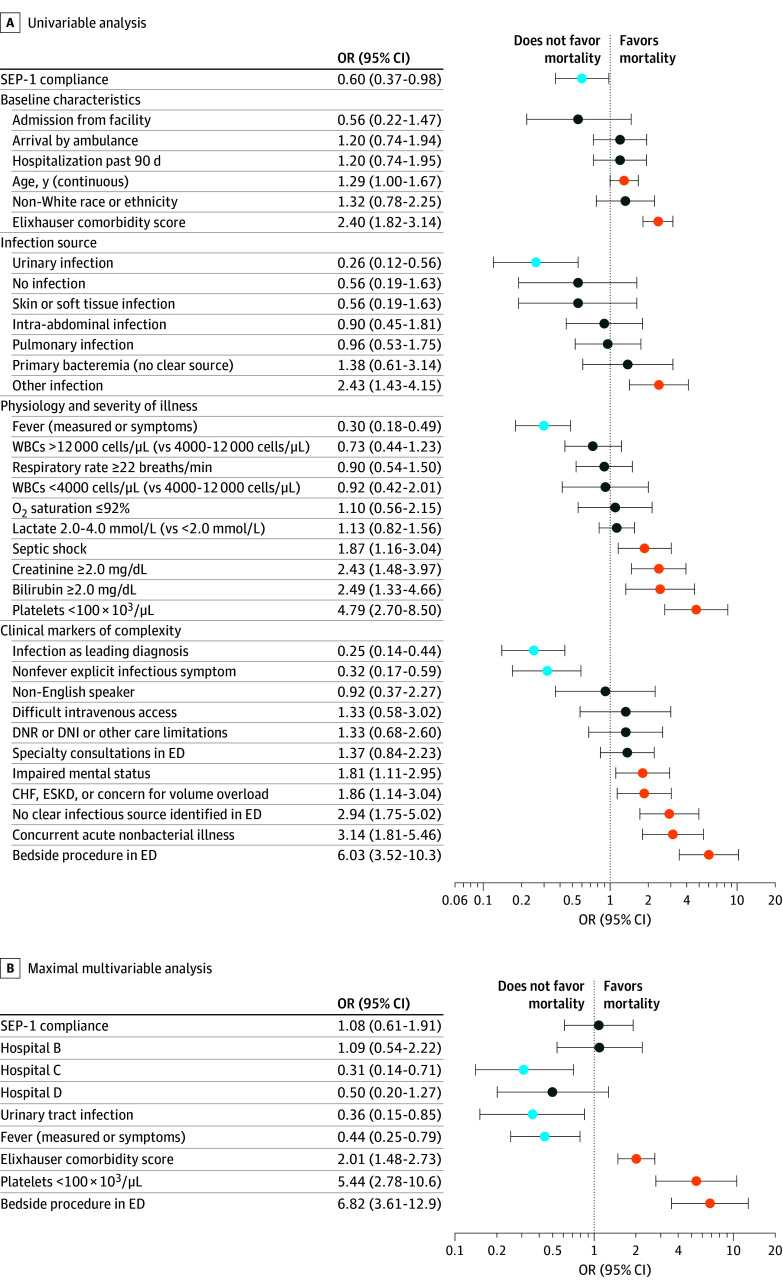
Univariable and Maximal Multivariable Analysis for Variables Associated With Hospital Mortality A, Univariable analysis. “Fever (measured or symptoms)” combines initial measured temperature in the emergency department (ED) or subjective fever, chills, or rigors obtained by history. B, Maximal multivariable analysis. Hospital A is the reference category for the other hospitals (B-D). Statistically significant variables associated with lower mortality are highlighted in light blue; statistically significant variables associated with higher mortality are highlighted in orange. CHF indicates congestive heart failure; DNI, do not intubate; DNR, do not resuscitate; ESKD, end-stage kidney disease; OR, odds ratio; SEP-1, Severe Sepsis and Septic Shock Management Bundle; and WBCs, white blood cells. SI conversion factors: To convert creatinine to micromoles per liter, multiply by 88.4; bilirubin to micromoles per liter, multiply by 17.104; platelets to cells ×10^9^/L, multiply by 1.0; and WBCs to cells ×10^9^/L, multiply by 0.001.

In-hospital mortality was lower among patients who received SEP-1–compliant care vs SEP-1–noncompliant care (40 of 335 [11.9%] vs 41 of 255 [16.1%]; OR, 0.60 [95% CI, 0.37-0.98]). Covariates associated with increased risk of in-hospital mortality on univariable analysis included age; AHRQ Elixhauser score; kidney dysfunction; hepatic dysfunction; thrombocytopenia; septic shock; impaired mental status; congestive heart failure, end-stage kidney disease, or documented concern for volume overload; need for bedside procedures; lack of clear infection source in the ED; and concurrent acute nonbacterial process. In contrast, urinary source of infection, fever (by initial measured temperature or by symptoms), and infection as the leading diagnosis were associated with lower risk of death (Figure 3A).

**Figure 4.**
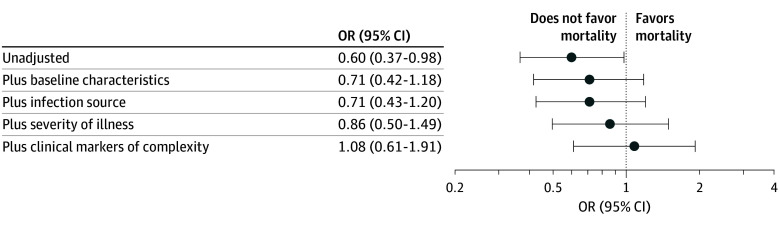
Association Between SEP-1 Compliance and In-Hospital Death in Multivariable Models Incorporating Successively Detailed Sets of Covariates Each successive model includes all the previous categories (ie, “Plus clinical markers of complexity” includes baseline characteristics, infection source, and severity of illness). OR indicates odds ratio; SEP-1, Severe Sepsis and Septic Shock Management Bundle.

The association between SEP-1 compliance and in-hospital mortality was attenuated after adjusting for demographics and comorbidities (unadjusted OR, 0.60 [95% CI, 0.37-0.98]; adjusted OR [AOR], 0.71 [95% CI, 0.42-1.18]), did not change when adding infection source (AOR, 0.71 [95%, CI 0.43-1.20]), and became further attenuated when adding severity of illness (AOR, 0.86 [95% CI, 0.50-1.49]) and clinical markers of complexity (AOR, 1.08 [95% CI, 0.61-1.91]) (Figure 4). All gVIF values were close to 1, indicating no strong evidence of multicollinearity, and no significant interaction effects were detected using type II and type III analysis of variance. In the maximal multivariable analysis, AHRQ Elixhauser score, thrombocytopenia, and need for bedside procedures were significantly associated with increased risk of mortality; fever and urinary tract infections were associated with lower risk of mortality (Figure 3B). Full model results are shown in eTable 2 in Supplement 1.

On stratifying the analyses by severe sepsis vs septic shock, there was no significant association between SEP-1 compliance and mortality in either subgroup, but adding more covariates changed point estimates for severe sepsis (unadjusted OR, 0.74 [95% CI, 0.37-1.49] to maximally adjusted OR, 1.73 [95% CI, 0.74-4.01]) but not for septic shock (unadjusted OR, 0.52 [95% CI, 0.25-1.05] to maximally adjusted OR, 0.53 [95% CI, 0.21-1.29]) (eFigure 3 in Supplement 1). Results were similar when using a composite outcome of in-hospital death, discharge to hospice, or ICU length of stay of 3 days or more for all sepsis cases (eFigure 4 in Supplement 1).

## Discussion

In this multicenter cohort study of 590 patients with sepsis, those who received care that was noncompliant with SEP-1 tended to be older, have more comorbidities, and more frequently presented with primary bloodstream infections of unclear source, septic shock, kidney dysfunction, and thrombocytopenia compared with those who received SEP-1–compliant care. They were also more likely to present with clinical syndromes with broad differential diagnoses and characteristics liable to complicate the diagnosis and management of sepsis, such as nonfebrile symptoms, impaired mental status, need for bedside procedures (particularly central venous catheters and intubation), and acute concurrent noninfectious conditions. Adjusting for these confounders, many obtainable only through detailed medical record review, shifted the effect estimate for the association between SEP-1 compliance and mortality from protective to null.

Our findings are consistent with other analyses documenting that patients who receive care that is noncompliant with SEP-1 are more likely to have septic shock, heart failure, and end-stage kidney disease and are less likely to present with fever compared with patients who receive care that is compliant.^16,17,18,21,22,29,30^ Our study extends these observations by identifying additional factors that complicate sepsis diagnosis and management and helps further clarify why some patients are less likely to receive SEP-1–compliant care (eg, more time is needed to establish a diagnosis, competing acute noninfectious issues require immediate management, and there is a greater need to titrate fluids and other interventions to patients’ clinical responses) and have higher mortality rates. The greater frequency of concurrent nonbacterial processes in the noncompliant group highlights the reality that sepsis can often present with other severe acute illnesses (eg, myocardial infarction, cardiogenic shock, arrhythmias, progressive cancer) that warrant immediate attention (thereby delaying sepsis-specific care) and worsen prognosis.^20,31,32,33^ Bedside procedures can consume time that delays other care; they are also likely markers of more severe illness. Impaired mental status may cause diagnostic delays due to challenges in obtaining a history, may slow the provision of care, and portend worse prognoses.^34,35^

Adjusting for the full breadth of confounders in our study, particularly after adding clinical markers of complexity, shifted point estimates for the association between SEP-1 compliance and mortality from protective to null. On subgroup analysis, this change was associated with patients with severe sepsis; point estimates did not change for patients with septic shock. Most prior studies that reported lower mortality rates with SEP-1 compliance adjusted only for the limited covariates available in administrative or EHR datasets, including a recent large propensity-matched analysis led by CMS that did not even match on severe sepsis vs septic shock.^5,10,36^ The lack of a significant protective association after detailed risk adjustment in our study may reflect the fact that only a small fraction of SEP-1–noncompliant cases (16.5% in our study) failed due to the 3-hour antibiotic requirement; prior studies have consistently demonstrated that timely antibiotic therapy is the most important bundle component, particularly for patients with septic shock.^5,16,36,37^

The high risk of residual confounding in observational studies comparing outcomes in SEP-1–compliant vs SEP-1–noncompliant patients underscores the value of using alternative study designs to evaluate the association of SEP-1 with outcomes. In the absence of randomized clinical trials, the next most rigorous option is interrupted time-series analyses evaluating the association of SEP-1’s implementation with temporal trends in mortality and other outcomes. Several time-series analyses have now been published which collectively suggest that implementation of SEP-1 in 2015 was associated with increases in broad-spectrum antibiotic use, lactate measurements, and 30-mL/kg fluid resuscitation but not with lower sepsis mortality rates.^38,39,40,41^

These studies and our current analysis raise concern that the CMS plan to change SEP-1 from pay for reporting to pay for performance may not catalyze meaningful gains in sepsis survival. Although there is room to improve bundle compliance rates (56.8% in our study and approximately 60% nationally in 2023^42^), our findings suggest that bundle noncompliance does not always reflect poor care but can reflect the complexity of some sepsis presentations and the frequent presence of other acute conditions that require simultaneous diagnosis and management. These controversies underlie recent recommendations by the Infectious Diseases Society of America and other professional societies to shift the focus of sepsis quality measures from early resuscitation bundles to risk-adjusted outcome metrics that will allow hospitals more flexibility to customize care for patients with complicated presentations and incentivize hospitals to pay more attention to the full breadth of sepsis care throughout hospitalization.^11,43^

### Strengths and Limitations

This study has some strengths. The major strengths are its multicenter design and use of detailed medical record reviews to glean novel insights into complex sepsis presentations that cannot be obtained from structured data alone.

Our study also has some limitations. First, we included only academic hospitals; the case mix, complexity, and management of patients presenting to community hospitals with sepsis may differ. Second, our sample size lacked sufficient power to detect statistically significant differences in some characteristics and outcomes, and the associations between SEP-1 compliance and outcomes must be interpreted with caution, as most point estimates did not reach statistical significance. In particular, patients with septic shock comprised only 36.3% of our sample; for this reason, we believe our findings are most relevant to patients without septic shock. Third, we were only able to abstract elements recorded in patients’ medical records, and some of the elements that we abstracted were potentially subjective. We attempted to mitigate this using a structured review process, an initial overlapped set of training cases, and regular multidisciplinary investigator meetings to discuss and adjudicate ambiguous scenarios. Fourth, we did not use a standardized severity-of-illness score (such as APACHE [Acute Physiology and Chronic Health Evaluation] II or SOFA [Sequential Organ Failure Assessment]) in our multivariable models. However, we adjusted for numerous individual physiological covariates that comprise these scores, an approach which can provide more granular insights into specific factors associated with sepsis outcomes.^44^ Fifth, we did not abstract data on the adequacy of empirical antibiotics relative to identified pathogens, an important mediator of sepsis outcomes.^45^ Sixth, we focused on sepsis presentations and management only in the ED; additional research is needed to understand whether our findings apply to patients who develop sepsis in inpatient settings.

## Conclusions

This cohort study found that patients with sepsis who receive SEP-1–noncompliant vs SEP-1–compliant care have significant differences in their clinical presentations, diagnostic complexity, and need for competing therapies that are poorly captured in most observational studies but likely confound the association between SEP-1 compliance and mortality. These findings have important implications for ongoing deliberations on how best to use national quality measures to catalyze improvements in sepsis outcomes.

## Notes

Supplement 1. **eMethods.** Additional Details About Data Collection and Medical Record Review Process **eTable 1.** SEP-1 Compliance Rates and Outcomes by Hospital Site **eTable 2.** Full Multivariable Model Results for Associations Between SEP-1 Compliance and Hospital Mortality **eTable 2a.** Selected Multivariable Model After Adding Baseline Characteristics **eTable 2b.** Selected Multivariable Model After Adding Infection Source **eTable 2c.** Selected Multivariate Model After Adding Physiologic Variables and Severity of Illness **eTable 2d.** Selected Multivariate Model After Adding Clinical Markers of Complexity **eFigure 1.** Distribution of Bedside Procedures in the Emergency Department **eFigure 2.** Distribution of Acute Concurrent Nonbacterial Illnesses **eFigure 3.** Association Between SEP-1 Compliance and In-Hospital Death in Univariable and Maximally Adjusted Multivariable Models for Severe Sepsis Cases Only (N=376) and Septic Shock Cases Only (N=214) **eFigure 4.** Association Between SEP-1 Compliance and Composite Outcome (In-Hospital Death, Discharge to Hospice, or ICU LOS ≥3 Days) in Multivariable Models Incorporating Successively Detailed Sets of Covariates (All Sepsis Cases, N=590) **eAppendix.** Chart Review Abstraction Tool

Supplement 2.
Data Sharing Statement
