# Supplementary material for: Complex Sepsis Presentations, SEP-1 Compliance, and Outcomes
Source: JAMA Netw Open. 2025 Mar 19;8(3):e251100. doi: 10.1001/jamanetworkopen.2025.1100 (PMC11923707; doi:10.1001/jamanetworkopen.2025.1100)
Supplement: Supplement 1. — eMethods. Additional Details About Data Collection and Medical Record Review Process eTable 1. SEP-1 Compliance Rates and Outcomes by Hospital Site eTable 2. Full Multivariable Model Results for Associations Between SEP-1 Compliance and Hospital Mortality eTable 2a. Selected Multivariable Model After Adding Baseline Characteristics eTable 2b. Selected Multivariable Model After Adding Infection Source eTable 2c. Selected Multivariate Model After Adding Physiologic Variables and Severity of Illness eTable 2d. Selected Multivariate Model After Adding Clinical Markers of Complexity eFigure 1. Distribution of Bedside Procedures in the Emergency Department eFigure 2. Distribution of Acute Concurrent Nonbacterial Illnesses eFigure 3. Association Between SEP-1 Compliance and In-Hospital Death in Univariable and Maximally Adjusted Multivariable Models for Severe Sepsis Cases Only (N=376) and Septic Shock Cases Only (N=214) eFigure 4. Association Between SEP-1 Compliance and Composite Outcome (In-Hospital Death, Discharge to Hospice, or ICU LOS ≥3 Days) in Multivariable Models Incorporating Successively Detailed Sets of Covariates (All Sepsis Cases, N=590) eAppendix. Chart Review Abstraction Tool [file jamanetwopen-e251100-s001.pdf]

Supplementary Online Content

Rhee C, Train SE, Filbin MR, et al. Complex sepsis presentations, SEP-1 compliance, and outcomes. *JAMA Netw Open.* 2025;8(3):e251100. doi:10.1001/jamanetworkopen.2025.1100

eMethods. Additional Details About Data Collection and Medical Record Review Process

eTable 1. SEP-1 Compliance Rates and Outcomes by Hospital Site

eTable 2. Full Multivariable Model Results for Associations Between SEP-1 Compliance and Hospital Mortality

eTable 2a. Selected Multivariable Model After Adding Baseline Characteristics

eTable 2b. Selected Multivariable Model After Adding Infection Source

eTable 2c. Selected Multivariate Model After Adding Physiologic Variables and Severity of Illness

eTable 2d. Selected Multivariate Model After Adding Clinical Markers of Complexity

eFigure 1. Distribution of Bedside Procedures in the Emergency Department

eFigure 2. Distribution of Acute Concurrent Nonbacterial Illnesses

eFigure 3. Association Between SEP-1 Compliance and In-Hospital Death in Univariable and Maximally Adjusted Multivariable Models for Severe Sepsis Cases Only (N=376) and Septic Shock Cases Only (N=214)

eFigure 4. Association Between SEP-1 Compliance and Composite Outcome (In-Hospital Death, Discharge to Hospice, or ICU LOS ≥3 Days) in Multivariable Models Incorporating Successively Detailed Sets of Covariates (All Sepsis Cases, N=590)

eAppendix. Chart Review Abstraction Tool

This supplementary material has been provided by the authors to give readers additional information about their work.

© 2025 Rhee C et al. *JAMA Network Open.*

eMethods. Additional Details About Data Collection and Medical Record Review Process

SEP-1 cases were obtained from each hospitals' quality officer responsible for CMS reporting at Brigham and Women's Hospital, Massachusetts General Hospital, University of Iowa Hospitals and Clinics, and University of California, Irvine Medical Center. Investigative teams at each hospital were then responsible for abstraction the data elements in the REDCap data collection tool.

For cases at Massachusetts General Hospital and Brigham and Women's Hospital, experienced analysts populated elements that were electronically available in the Enterprise Data Warehouse (marked in the REDCap form as "electronically extractable"). For the cases at Massachusetts General Hospital, college graduate level clinical research assistants experienced in sepsis cohort enrollment and data extraction manually abstracted other objective elements of the REDCap form, then an experienced emergency medicine clinical pharmacist and emergency physician abstracted cases for the detailed clinical questions about patients' presentation and ED course. At Brigham and Women's Hospital, all questions that were not electronically populated were abstracted by fellows in Infectious Diseases and Pulmonary/Critical Care Medicine. For UC Irvine and University of Iowa, all data elements were manually abstracted by the investigative team, which included Emergency Medicine clinical pharmacists and attending physicians in Emergency Medicine and Infectious Diseases.

All datasets were stripped of identifiers, collated by the coordinating team at Harvard Pilgrim Health Care Institute, and then inspected by analysts and the principal investigator for completeness. Any missing data identified during the data collation phase were backfilled by the investigators at each site, resulting in no missing data elements in the final analytic dataset.

At each site, an initial 15 cases were independently reviewed by at least two reviewers and the key clinical aspects were discussed and resolved amongst the reviewers to ensure a standardized process moving forward. Afterwards, reviewers completed each case review independently. Cases for which there were questions about how to abstract data elements were flagged and brought to monthly investigator meetings for group discussion and adjudication.

© 2025 Rhee C et al. *JAMA Network Open.*

eTable 1. SEP-1 Compliance Rates and Outcomes by Hospital Site

Hospital	SEP-1 Compliance	In-Hospital Death	Death, Discharge to Hospice, or ICU Admission ≥3 Days
Hospital A	91 / 150 (60.7%)	28 / 122 (18.7%)	55 / 150 (36.7%)
Hospital B	98 / 147 (66.7%)	30 / 147 (20.4%)	54 / 147 (36.7%)
Hospital C	67 / 143 (46.9%)	14 / 143 (9.8%)	45 / 143 (31.5%)
Hospital D	79 / 150 (52.7%)	9 / 150 (6.0%)	46 / 150 (30.7%)

© 2025 Rhee C et al. *JAMA Network Open.*

eTable 2. Full Multivariable Model Results for Associations Between SEP-1 Compliance and Hospital Mortality

The following tables show the intermediate and final multivariable models for the associations between SEP-1 compliance and hospital mortality incorporating successively complex sets of covariates. The models were selected by BIC (Bayesian information criteria) using a forward-backward stepwise search algorithm. The algorithm searches between a specified minimal model, which is the model selected in the previous layer, and a specified maximal model, which consists of the previously selected model plus all new predictors. To check the robustness and performance of the selected models, we calculated cross-validated area under the curves (AUCs) by repeating 10-fold cross validation 100 times (denoted as cv-AUC). All the cv-AUC values are close to the original model's AUC values, suggesting that these models are likely not overfitting the data and their performances are consistent and robust across different subsets of data.

At layer 1, the model only includes SEP-1 compliance and each individual hospital as predictors as in univariable association analysis (BIC=480.57, AUC = 0.66, cv-AUC=0.65).

eTable 2a. Selected Multivariable Model After Adding Baseline Characteristics (Layer 2: BIC=443.82, AUC=0.76, cv-AUC=0.75)

Predictor	Odds Ratio	Lower 95% CI	Upper 95% CI	p-value
(Intercept)	0.18	0.10	0.31	<0.001
SEP-1 Compliance	0.71	0.42	1.18	0.184
Hospital B (vs A)	1.52	0.82	2.84	0.183
Hospital C (vs A)	0.37	0.18	0.77	0.008
Hospital D (vs A	0.65	0.27	1.55	0.329
Elixhauser Comorbidity Score	2.35	1.79	3.09	<0.001

eTable 2b. Selected Multivariable Model After Adding Infection Source (Layer 3, BIC=440.12, AUC=0.78, cv-AUC=0.76)

Predictor	Odds Ratio	Lower 95% CI	Upper 95% CI	p-value
(Intercept)	0.21	0.12	0.38	<0.001
SEP-1 Compliance	0.71	0.43	1.20	0.2.00
Hospital B (vs A)	1.55	0.83	2.91	0.171
Hospital C (vs A)	0.41	0.19	0.86	0.018
Hospital D (vs A	0.64	0.27	1.53	0.314
Elixhauser Comorbidity Score	2.28	1.72	3.00	<0.001
Urinary Source of Infection	0.32	0.14	0.70	0.004

© 2025 Rhee C et al. *JAMA Network Open.*

eTable 2c. Selected Multivariate Model After Adding Physiologic Variables and Severity of Illness
(Layer 4: BIC=425.87, AUC=0.82, cv-AUC=0.80)

Predictor	Odds Ratio	Lower 95% CI	Upper 95% CI	p-value
(Intercept)	0.24	0.12	0.46	<0.001
SEP-1 Compliance	0.86	0.50	1.49	0.599
Hospital B (vs A)	1.61	0.83	3.12	0.161
Hospital C (vs A)	0.41	0.19	0.88	0.023
Hospital D (vs A	0.59	0.24	1.44	0.244
Elixhauser Comorbidity Score	1.98	1.48	2.64	<0.001
Urinary Source of Infection	0.34	0.15	0.76	0.009
Thrombocytopenia	3.92	2.11	7.30	<0.001
Fever (Measured or by Symptoms)	0.38	0.22	0.66	0.001

eTable 2d. Selected Multivariate Model After Adding Clinical Markers of Complexity
(Layer 5: BIC=396.67, AUC=0.87, cv-AUC=0.85)

Predictor	Odds Ratio	Lower 95% CI	Upper 95% CI	p-value
(Intercept)	0.13	0.06	0.27	<0.001
SEP-1 Compliance	1.08	0.61	1.91	0.803
Hospital B (vs A)	1.09	0.54	2.22	0.807
Hospital C (vs A)	0.31	0.14	0.71	0.005
Hospital D (vs A	0.50	0.20	1.27	0.147
Elixhauser Comorbidity Score	2.01	1.48	2.73	<0.001
Urinary Source of Infection	0.36	0.15	0.85	0.020
Thrombocytopenia	5.44	2.78	10.64	<0.001
Fever (Measured or by Symptoms)	0.44	0.25	0.79	0.006
Bedside Procedure in the ED	6.82	3.61	12.89	<0.001

© 2025 Rhee C et al. *JAMA Network Open.*

eFigure 1. Distribution of Bedside Procedures in the Emergency Department

The listed percentages are relative to all sepsis cases in the cohort (n=590).

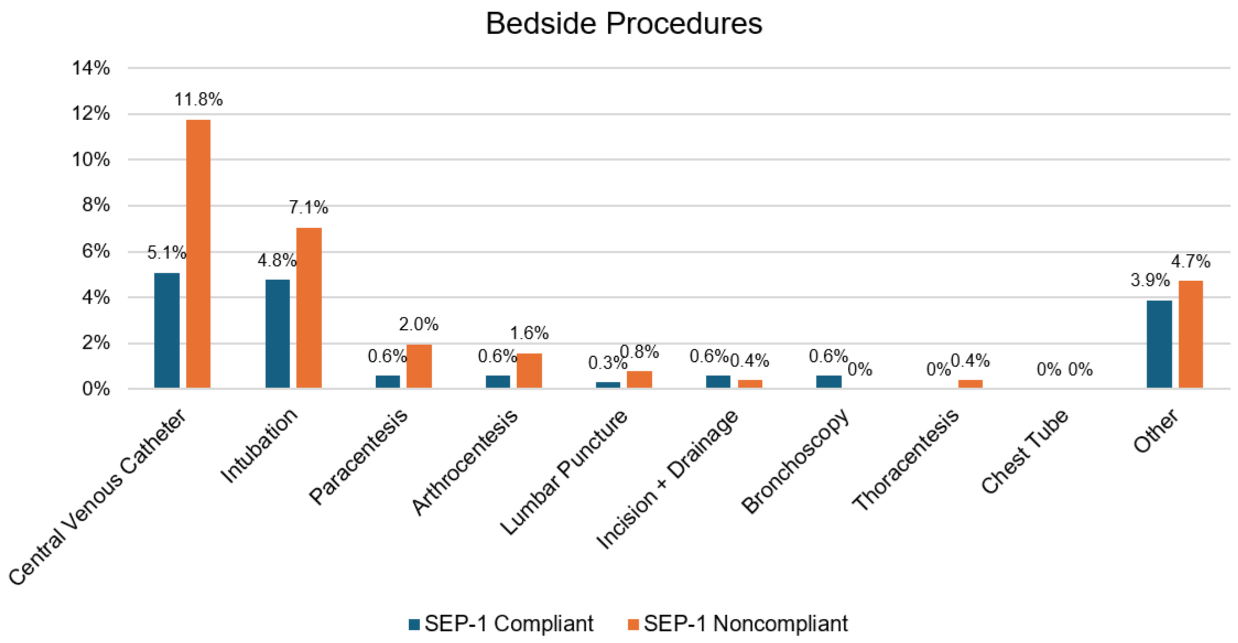

© 2025 Rhee C et al. *JAMA Network Open.*

eFigure 2. Distribution of Acute Concurrent Nonbacterial Illnesses

The listed percentages are relative to the cases that had an acute non-bacterial condition (n=255).

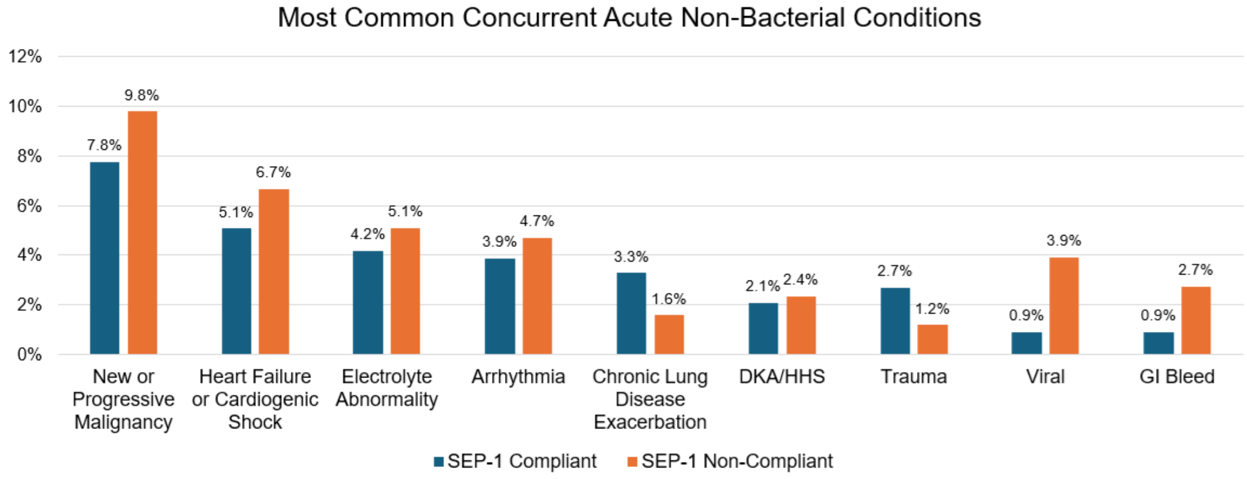

© 2025 Rhee C et al. *JAMA Network Open.*

eFigure 3. Association Between SEP-1 Compliance and In-Hospital Death in Univariable and Maximally Adjusted Multivariable Models for Severe Sepsis Cases Only (N=376) and Septic Shock Cases Only (N=214)

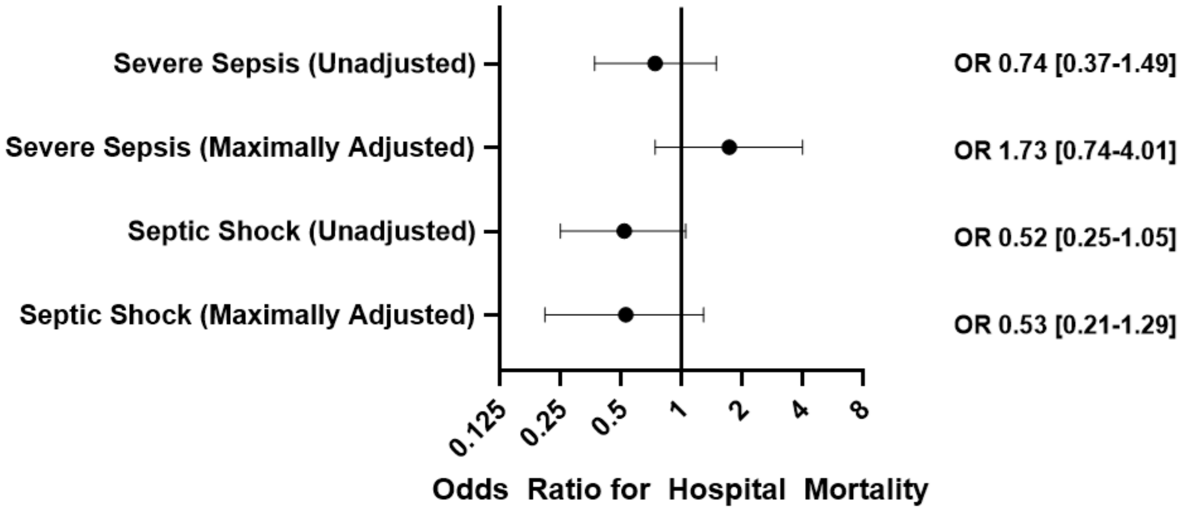

© 2025 Rhee C et al. *JAMA Network Open.*

eFigure 4. Association Between SEP-1 Compliance and Composite Outcome (In-Hospital Death, Discharge to Hospice, or ICU LOS ≥3 Days) in Multivariable Models Incorporating Successively Detailed Sets of Covariates (All Sepsis Cases, N=590)

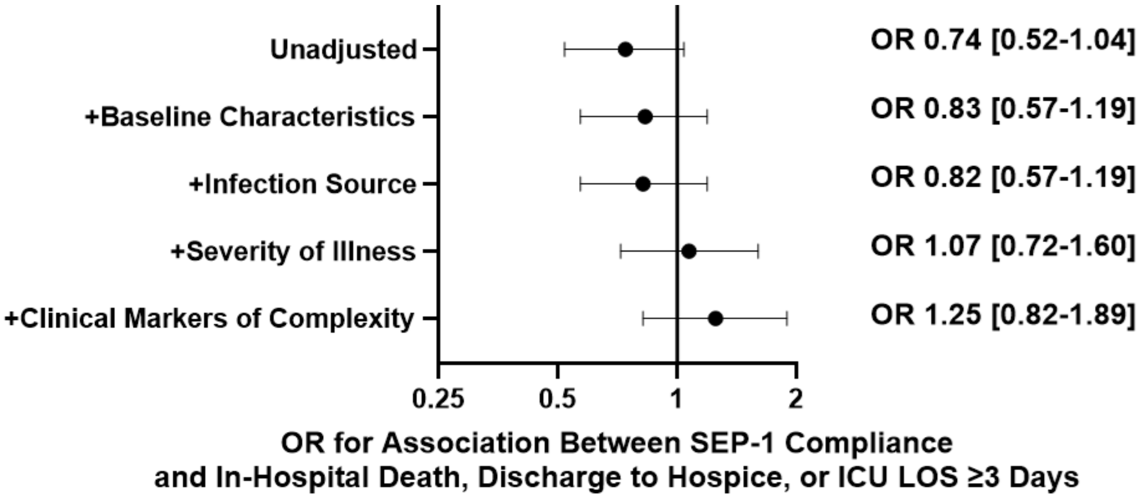

© 2025 Rhee C et al. *JAMA Network Open.*

eAppendix. Chart Review Abstraction Tool

BACKGROUND AND ENCOUNTER INFORMATION	
Patient MRN/ID	______________________
Hospital	◯ BWH<br>◯ MGH<br>◯ Iowa<br>◯ UC Irvine<br>◯ Other
Date/Time of ED Arrival (M-D-Y H:M)	______________________
Date/Time of ED Departure (M-D-Y H:M)<br><br>(This is when the patient physically leaves the ED. The date should also correspond to the hospital admission date.)	______________________
Did the patient board as an inpatient while in the ED?	Yes<br>No
Time patient became an ED boarder (M-D-Y H:M)	______________________
Date of Hospital Discharge	______________________
Patient's Age (years)<br><br>*Note: this data element can be electronically extracted*	______________________
Race<br><br>*Note: this data element can be electronically extracted*	◯ White<br>◯ Black<br>◯ Asian<br>◯ American Indian / Alaska Native<br>◯ Native Hawaiian or Other Pacific<br>◯ Two or more races<br>◯ Unknown
Ethnicity<br><br>*Note: this data element can be electronically extracted*	◯ Not Hispanic/Latino<br>◯ Hispanic/Latino

© 2025 Rhee C et al. *JAMA Network Open.*

How did the patient arrive in the ED?	◯ Walk-in/Self-referral<br>◯ EMS<br>◯ Sent in from clinic<br>◯ Other
Preadmission location/status	◯ Home (Community)<br>◯ Assisted Living<br>◯ Long-Term or Subacute Care Facility<br>◯ Psychiatric Facility<br>◯ Hospice Facility<br>◯ Home Hospice<br>◯ Other
Other preadmission location	______________________
ED Discharge Disposition	◯ Home<br>◯ ED Observation Unit<br>◯ Inpatient non-ICU ward<br>◯ ICU<br>◯ Palliative Care Unit<br>◯ Transfer to Another Acute Care Hospital<br>◯ Transfer to Non-Acute Facility<br>◯ Death<br>◯ Other
Admitting Service	◯ Medical (including hematology/oncology, cardiology, medical ICU, or COVID units)<br>◯ Surgical (including SICU and surgical subspecialties)<br>◯ Obstetrics/Gynecology<br>◯ Neurology (including neuro-ICU)<br>◯ Psychiatry<br>◯ Palliative Care<br>◯ Other
Inpatient hospitalization with date of discharge within the past 90 days?<br><br>*Note: this data element can be electronically extracted. It is acceptable if this misses some outside hospital discharges if the EHR only contains information on hospitalizations within the same healthcare system).	◯ Yes<br>◯ No
Date of last hospital discharge within 90 days	______________________
ICU length of stay (calendar days; count all ICU days during entire hospitalization, including from multiple ICU admissions if applicable. If no ICU admission, enter 0)<br><br>*Note: this data element can be electronically extracted*	______________________

© 2025 Rhee C et al. *JAMA Network Open.*

First IV Antibiotic Administered<br><br>*Note: this data element can be electronically extracted*	◯ Amikacin<br>◯ Aztreonam<br>◯ Cefepime<br>◯ Cefiderocol<br>◯ Cefotaxime<br>◯ Ceftriaxone<br>◯ Ceftazidime<br>◯ Ceftazidime-Avibactam<br>◯ Ceftolozane-Tazobactam<br>◯ Ciprofloxacin<br>◯ Daptomycin<br>◯ Ertapenem<br>◯ Gentamicin<br>◯ Imipenem<br>◯ Imipenem-Relebactam<br>◯ Levofloxacin<br>◯ Linezolid<br>◯ Meropenem<br>◯ Meropenem-Vaborbactam<br>◯ Moxifloxacin<br>◯ Piperacillin-Tazobactam<br>◯ Tedizolid<br>◯ Tobramycin<br>◯ Vancomycin<br>◯ Other
Other first IV antibiotic	____________________
Date/Time of First IV Antibiotic Administration<br><br>*Note: this data element can be electronically extracted*	____________________
Hospital Discharge Disposition<br><br>*Note: this data element can be electronically extracted*	◯ Home<br>◯ Hospice Home<br>◯ Hospice Health Care Facility<br>◯ Transfer to Acute Care Hospital<br>◯ Transfer to Intermediate/Long-Term Care Facility<br>◯ Transfer to Psychiatric Facility<br>◯ Expired<br>◯ Left AMA<br>◯ Not Documented, Unable to Determine

**SEP-1 SPECIFIC INFORMATION (FROM HOSPITAL'S QUALITY OFFICER)**	
Sepsis Time Zero per SEP-1 Abstractor (M-D-Y H:M)<br><br>Note: please ensure time zero occurred in the ED; if not, please stop abstracting.	____________________
Did the patient have initial hypotension as part of severe sepsis criteria? (This should be included as a discrete field within the SEP-1 abstraction report).	◯ No - no initial hypotension documented<br>◯ Yes - initial hypotension documented
Did the patient meet CMS criteria for Septic Shock or only Severe Sepsis (according to the hospital's SEP-1 abstractor)?	◯ Severe Sepsis<br>◯ Septic Shock

© 2025 Rhee C et al. *JAMA Network Open.*

Did the case pass or fail SEP-1?	◯ Pass<br>◯ Fail
If the case failed, what element did the case fail on? (Check the first failed element in the SEP-1 pathway.)	◯ Initial lactate (3 hour bundle)<br>◯ Blood culture before antibiotics (3 hour bundle)<br>◯ Broad spectrum antibiotics (3 hour bundle)<br>◯ 30 cc/kg fluids (3 hour bundle, for initial hypotension or lactate ≥4.0 mmol/L)<br>◯ Repeat lactate (6 hour bundle)<br>◯ Vasopressors (6 hour septic shock bundle)<br>◯ Repeat volume / perfusion assessment (6 hour septic shock bundle)<br>◯ Other (specify)
Other reason for SEP-1 failure (free text)	____________________

DISCHARGE DIAGNOSIS CODES

These can be electronically extracted

Principal Diagnosis ICD-10 code (no decimal points)<br><br>Examples: A4151, T8579XA, C786, M4802, etc. (only include a single code)	____________________
Secondary Diagnosis ICD-10 Codes<br><br>Enter ALL secondary codes. Separate each code by a semicolon.<br><br>For each code, include a "1" in parentheses it is present-on-admission (POA), or "0" if not POA.<br><br>Example:<br>E1110(1); B004(1); A419(0), I471(0), E872(0), Z68(0), E46(0), J981(0), F19239(0)	____________________

SEVERITY OF ILLNESS IN ED

Note: all of the data elements in this section can be electronically extracted

Initial Temperature Value (Farenheit)	____________________
Initial Systolic Blood Pressure Value	____________________
Initial Respiratory Rate Value	____________________
Initial O2 Sat Value	____________________

© 2025 Rhee C et al. *JAMA Network Open.*

Initial O2 Device Support	◯ None (room air)<br>◯ Simple nasal cannula (< 3 L)<br>◯ Simple nasal cannula (>=3 L)<br>◯ Oxymizer<br>◯ Face mask<br>◯ Non-rebreather<br>◯ High flow oxygen<br>◯ Non-invasive ventilation<br>◯ Invasive mechanical ventilation
Initial Lactate Date/Time	______________________
Initial Lactate level (mmol/L)	______________________
Initial Creatinine Date/Time	______________________
Initial Creatinine Value (mg/dL)	______________________
Initial Total Bilirubin Date/Time	______________________
Initial Total Bilirubin Value (mg/dL) (leave blank if missing)	______________________
Initial Platelet Count Date/Time	______________________
Initial Platelet Value (10^9/L, normal range = 150-400)	______________________
Initial WBC Date/Time	______________________
Initial WBC Value (10^9/L, normal range = 4.0-10.0)	______________________
Hypotension (SBP < 90 mmHg) while in the ED?	◯ Yes<br>◯ No
Vasopressors while in the ED?	◯ Yes<br>◯ No
Highest O2 Device while in ED	◯ None (room air)<br>◯ Simple nasal cannula (< 3 L)<br>◯ Simple nasal cannula (>=3 L)<br>◯ Oxymizer<br>◯ Face mask<br>◯ Non-rebreather<br>◯ High flow oxygen<br>◯ Non-invasive ventilation<br>◯ Invasive mechanical ventilation

© 2025 Rhee C et al. *JAMA Network Open.*

**CLINICAL PRESENTATION AND COURSE**<br><br>***Note: all of these require MANUAL abstraction by chart review***	
Were any of the following potential barriers to care present? Check all that apply.<br><br>These can be gleaned from ED notes and/or admitting H+P or other sources in the medical record.	☐ Alcohol or drug intoxication<br>☐ Aggressive Behavior<br>☐ Altered mental status /Delirium<br>☐ Dementia history<br>☐ Difficult IV access<br>☐ Non-English Speaker<br>☐ Opioid Dependence<br>☐ Poor Historian (as documented by providers). (Note: this refers to being a poor historian in the absence of AMS or dementia).<br>☐ Refusing any aspect of care<br>☐ None of the above barriers present
Issues with difficult IV access (check all that apply)<br><br>This can be gleaned from ED notes, procedure notes, as well as ED nursing notes.	☐ Multiple IV attempts documented<br>☐ Need for ultrasound-guided peripheral IV<br>☐ Need for IO<br>☐ Need for central line placement<br>☐ Other
Was there documentation of the presence of a support person (i.e., spouse, family member, etc.) in the ED?	○ Yes<br>○ No
Was the patient DNR/DNI or have other limitations in care in the ED? Check all that apply	☐ DNR or DNI<br>☐ No ICU / escalation of care<br>☐ Comfort measures only<br>☐ Other limitations in care<br>☐ No limitations in care (i.e., full code and full aggressive care)
Did the patient present with explicit infectious symptoms? Check all that apply.<br><br>Please infer this from chief complaint, HPI, ED notes, and include signs/symptoms prior to ED arrival or identified on ED arrival/triage.<br><br>DO NOT include symptoms that were not present on arrival and only develop later in the ED course, for example fever that only manifests later during ED stay.	☐ Constitutional: fevers, chills, or rigors<br>☐ Respiratory: productive cough<br>☐ Urinary: dysuria, cloudy or foul-smelling urine<br>☐ Skin/Soft Tissue/Joint: skin or wound or joint redness, abscess, drainage<br>☐ Referral to ED for known or suspected infectious diagnosis<br>☐ Other explicit symptoms<br>☐ No explicit symptoms (i.e., presented with vague symptoms only)
Describe other explicit symptoms	______________________
The patient did not present to the ED with a history of or documented fevers or other explicit symptoms. Did the patient develop a fever (temp >38.0 C or 100.4 F) later in his/her ED course?	○ Yes<br>○ No
Did the patient have a history of congestive heart failure or end-stage renal disease?	☐ No ESRD or CHF<br>☐ Heart Failure<br>☐ ESRD
Was there documented concern by the ED providers for volume overload?	○ Yes<br>○ No

Did the patient require consultation to other specialties while in the ED? This includes curbsides and anyone who wrote a note, even if not physically seen in-person. Check all that apply	☐ No<br>☐ Yes - Medical Specialty<br>☐ Yes - Surgical Specialty<br>☐ Yes - Interventional Radiology<br>☐ Yes - Neurology<br>☐ Yes - Psychiatry<br>☐ Yes - Obstetrics/Gynecology<br>☐ Yes - Other
List the medical specialties consulted in the ED	☐ Allergy/Immunology<br>☐ Cardiology<br>☐ Endocrinology<br>☐ Gastroenterology/Hepatology<br>☐ Hematology<br>☐ Infectious Disease<br>☐ Nephrology<br>☐ Oncology<br>☐ Pulmonary/Critical Care<br>☐ Rheumatology<br>☐ Other IM Subspecialty
List the surgical specialties consulted in the ED	☐ Colorectal Surgery<br>☐ General/Trauma Surgery<br>☐ Otolaryngology<br>☐ Neurosurgery<br>☐ Plastic Surgery<br>☐ Ophthalmology<br>☐ Orthopedics<br>☐ Thoracic Surgery<br>☐ Cardiac Surgery<br>☐ Transplant Surgery<br>☐ Surgical Oncology<br>☐ Vascular Surgery<br>☐ Other Surgical Specialty
Radiology diagnostic procedures performed in the ED (check all that apply)	☐ X-ray<br>☐ CT scan<br>☐ MRI<br>☐ Ultrasound<br>☐ Other<br>☐ No radiology tests in ED
What type of x-ray was obtained?	☐ Chest<br>☐ Abdomen<br>☐ Soft Tissue/Bone/Joint<br>☐ Other
What type of CT scan was obtained?	☐ Head<br>☐ Chest<br>☐ Abd/Pelvis<br>☐ Extremity<br>☐ Other
What type of MRI was obtained?	☐ Brain<br>☐ Spine/Bone/Joint<br>☐ Abdomen<br>☐ Other

© 2025 Rhee C et al. *JAMA Network Open.*

What type of ultrasound was obtained?	☐ Lungs<br>☐ Abdomen/Pelvis/Vaginal<br>☐ Cardiac (including informal bedside TTE by ED provider)<br>☐ Other
Bedside procedures performed in the ED (check all that apply)	☐ Arthrocentesis<br>☐ Bronchoscopy<br>☐ Central Line Placement<br>☐ Chest Tube<br>☐ Incision and Drainage<br>☐ Intubation<br>☐ Lumbar Puncture<br>☐ Paracentesis<br>☐ Thoracentesis<br>☐ Other<br>☐ No bedside procedures
Did the patient require emergent IR or surgical procedure (i.e., transfer directly from the ED to the IR suite or the OR)?	◯ No<br>◯ Yes - surgery<br>◯ Yes - IR procedure
Was sepsis (including "sepsis", "severe sepsis", "septic shock") explicitly documented on the differential diagnosis of the ED providers (this may include residents, PAs, NPs, or attending providers)?<br><br>Please focus on documentation from ED providers, not the admitting team.	◯ Yes<br>◯ No
Was sepsis or infection considered to be the leading or most likely diagnosis or etiology for the patient's presentation in the ED (based on notes from ED residents, PAs, NPs, or attending providers)?<br><br>Please focus on documentation from ED providers, not the admitting team.	◯ Yes - sepsis felt to be the most likely etiology<br>◯ Yes - infection felt to be the most likely, but sepsis not explicitly suspected or documented (or if mentioned, sepsis felt less likely than infection without sepsis)<br>◯ No - a non-infectious process felt to be more likely
Was there a clear source of infection identified while in the ED that was apparent by the end of the patient's ED course?<br><br>For this question, you may use information from the admitting team's H+P as well as ED provider notes.	◯ Yes - clear source of infection identified in ED (e.g. pneumonia identified on chest radiograph with compatible symptoms; positive UA with compatible symptoms and/or imaging; skin/soft tissue infection, etc.)<br>◯ No - one or more sources may have been suspected but were not confirmed or clear while in the ED

© 2025 Rhee C et al. *JAMA Network Open.*

What was the primary source of infection responsible for the patient's presentation?<br><br>This takes into account all available information during the patient's hospitalization and beyond.<br><br>Choose one answer; if there were multiple potential sources, please choose the most likely or dominant source.	◯ Pulmonary<br>◯ Urinary<br>◯ Gastrointestinal or Intraabdominal<br>◯ Central Nervous System<br>◯ Skin/Soft Tissue<br>◯ Bone/Joint<br>◯ Vascular (i.e., Line, Endocarditis, Cardiac Device)<br>◯ Sinus<br>◯ Primary bacteremia (including oral/gut translocation, or bacteremia of unknown source)<br>◯ Febrile neutropenia but no clear source or organism identified<br>◯ Unknown<br>◯ Other<br>◯ Multiple sources<br>◯ No infection in retrospect
Did the patient have other acute non-bacterial conditions present on admission that may have contributed to the patient's presenting illness?	◯ Yes<br>◯ No
Other Acute Conditions (check all that apply)	☐ Viral, Fungal, or Parasitic Infection<br>☐ Cardiac Disease<br>☐ Pulmonary Disease<br>☐ Gastrointestinal Disease<br>☐ Neurologic Disease<br>☐ Endocrine Disease<br>☐ Hematologic/Oncology Disease<br>☐ Rheumatologic/Autoimmune Disease<br>☐ Renal<br>☐ Drugs/Toxins<br>☐ Other Miscellaneous
Specific Viral, Fungal, or Parasitic Infection	☐ SARS-CoV-2<br>☐ Influenza<br>☐ RSV<br>☐ Adenovirus<br>☐ Parainfluenza<br>☐ Human metapneumovirus<br>☐ Rhinovirus<br>☐ Presumed viral infection (no specific virus identified)<br>☐ Other virus<br>☐ Candida<br>☐ Mold<br>☐ Pneumocystis<br>☐ Other Fungal<br>☐ Parasitic
Specific Cardiac Disease	☐ Arrhythmia<br>☐ Heart failure / Volume overload (including Pulmonary edema)<br>☐ Cardiogenic shock<br>☐ Myocardial infarction or ischemia<br>☐ Myocarditis<br>☐ Valvular disease<br>☐ Other cardiac

Specific Pulmonary Disease	☐ ARDS<br>☐ Aspiration pneumonitis (only include if overt macro-aspiration, i.e. witnessed vomiting leading to pneumonitis)<br>☐ Exacerbation of chronic lung disease (asthma, bronchiectasis, COPD, ILD)<br>☐ Hypersensitivity pneumonitis<br>☐ Pulmonary embolism<br>☐ Other pulmonary
Specific GI disease	☐ Acute liver failure<br>☐ Alcoholic hepatitis<br>☐ Bowel obstruction<br>☐ GI bleed<br>☐ Hepatic encephalopathy<br>☐ Inflammatory bowel disease<br>☐ Mesenteric ischemia<br>☐ Pancreatitis<br>☐ Volvulus<br>☐ Other GI disease
Specific Neurologic Disease	☐ Autonomic dysfunction<br>☐ Seizure<br>☐ Stroke / Intracranial hemorrhage<br>☐ Heat stroke<br>☐ Other neurologic disease
Specific Endocrine Disease	☐ Adrenal insufficiency<br>☐ Diabetic ketoacidosis / hyperosmolar hyperglycemia nonketotic coma<br>☐ Hypoglycemia<br>☐ Hypothyroidism<br>☐ Hyperthyroidism<br>☐ Other endocrine disease
Specific Heme/Onc Disease	☐ Antiphospholipid syndrome<br>☐ New malignancy<br>☐ Progression of known malignancy<br>☐ Hemophagocytic syndrome<br>☐ Tumor lysis syndrome<br>☐ Other heme/onc process
Specific Rheumatologic/Autoimmune Disease	☐ Gout<br>☐ Rheumatoid arthritis<br>☐ Still's disease<br>☐ SLE<br>☐ Vasculitis<br>☐ Other rheumatologic/autoimmune disease
Specific Renal Disease	☐ Acute kidney injury<br>☐ Nephritic or nephrotic syndrome<br>☐ Electrolyte abnormality<br>☐ Volume overload related to renal failure (e.g., missed dialysis)<br>☐ Other renal disease

© 2025 Rhee C et al. *JAMA Network Open.*

Specific Drug/Toxin Effect	☐ Drug overdose<br>☐ Drug or alcohol withdrawal<br>☐ Hypersensitivity drug reaction (including anaphylaxis)<br>☐ Illicit drug effect<br>☐ Medication toxicity<br>☐ Malignant hyperthermia<br>☐ Neuroleptic malignant syndrome<br>☐ Serotonin syndrome<br>☐ Other drug/toxin effect
Other Miscellaneous Process	☐ Hypovolemia<br>☐ Hemorrhage (non-GI)<br>☐ Post-surgical inflammation<br>☐ Burns<br>☐ Trauma<br>☐ Allograft Rejection<br>☐ Other
Was bacterial infection or one of the above conditions the most likely driver of the patient's presenting syndrome?	◯ Bacterial Infection<br>◯ Non-Bacterial Syndrome (including viral/fungal infections or non-infectious etiologies)<br>◯ Both likely equally important, or unable to determine

CASE SUMMARY

Please provide a brief summary of the patient's course, with focus on the following factors:<br><br>- Explicit vs vague presenting symptoms<br>- Whether infection/sepsis was considered the most likely etiology of the patient's presentation in the ED, and if the source was clear<br>- Whether there other non-infectious processes contributing to the patient's presentation, and if so were these the primary contributor or secondary<br>- Any obstacles to sepsis recognition or sepsis care	______________________________
Does this case need to be flagged for additional discussion and review?	◯ Yes<br>◯ No

© 2025 Rhee C et al. *JAMA Network Open.*
