# Supplementary material for: Complex Sepsis Presentations, SEP-1 Compliance, and Outcomes
Source: JAMA Netw Open. 2025 Mar 19;8(3):e251100. doi: 10.1001/jamanetworkopen.2025.1100 (PMC11923707; doi:10.1001/jamanetworkopen.2025.1100)
Supplement: Supplement 2. — Data Sharing Statement [file jamanetwopen-e251100-s002.pdf]

# Data Sharing Statement

Rhee. Complex Sepsis Presentations, SEP-1 Compliance, and Outcomes. *JAMA Netw Open*.
Published March 19, 2025. doi:10.1001/jamanetworkopen.2025.1100

**Data**
**Data available:** No
